# Artificial intelligence assisted virtual reality training module for Gasserian ganglion block

**DOI:** 10.1016/j.inpm.2024.100536

**Published:** 2025-01-06

**Authors:** Oranicha Jumreornvong, David Chang, Gonzalo Povea Galdo, Jason Yong, Rohan Jotwani

**Affiliations:** a Department of Human Performance and Rehabilitation, Icahn School of Medicine at Mount Sinai, New York, NY, USA; b Department of Anesthesiology, Perioperative, and Pain Medicine, Icahn School of Medicine at Mount Sinai, New York, NY, USA; c Department of Biomedical Engineering, Universidad Peruana Cayetano Heredia, San Martin de Porres, Peru; d Department of Anesthesiology, Perioperative, and Pain Medicine, Harvard Medical School, Brigham and Women's Hospital, Boston, MA, USA; e Department of Anesthesiology, Perioperative, and Pain Medicine, Weill Cornell Medicine, New York, NY, USA

## Abstract

• **Immersive Learning Experience**: The AI-assisted VR module enables learners to engage in a 360-degree immersive environment, manipulating holographic anatomy models and simulating fluoroscopic guidance to perform the Gasserian ganglion block.
• **Anatomical Precision**: Key anatomical landmarks, like the foramen ovale, are highlighted, and proper C-arm positioning is demonstrated, helping practitioners localize the target area for needle advancement.
• **Multilingual and Multiple-Choice Support**: The module includes AI-driven multi-language options and AI-generated multiple-choice questions to enhance learning and retention.
• **GlobalScalability**: The proof of concept highlights the potential for scalable, remote learning solutions that integrate AI for ease of development and adaptation across diverse training environments worldwide.

Abstract

Gasserian ganglion block is a targeted procedure for managing severe facial pain, particularly in cases of trigeminal neuralgia and neuropathy. This proof-of-concept module presents a new type of pedagogy to teach advanced interventional pain procedures through the design and deployment of an Artificial Intelligence (AI)-assisted Virtual Reality (VR) training environment for learning the Gasserian ganglion block procedure. Through a 360° immersive experience, learners use VR headsets to explore and interact with a holographic anatomy models with narration and labels available in multiple languages. While the module includes anatomical guidance to recognize the foramen ovale (FO) and related landmarks, the primary goal is to correctly position the needle using intermittent fluoroscopic views. AI leverages advanced algorithms like deep learning and generative adversarial network to perform tasks requiring human intelligence. We applied these AI-driven 3D modeling techniques to rapidly create and edit simulated C-arm motion and AI-generated fluoroscopic models, guiding learners how to manipulate fluoroscopic views. We integrated it with VR anatomical sandbox software, 3D organon, and AI voice narration technology, utilizing advanced text analysis to synthesize natural tone and pitch through deep learning. The AI voice narration software translates written text into realistic speech, with capabilities for real-time subtitles and multi-language support. This guided narration facilitates step-by-step learning of critical procedures, from patient positioning to needle advancement toward the FO, with fluoroscopic checkpoints—such as lateral views—to ensure critical structures, like the clivus bone, remain intact. This module utilizes natural language processing and large language models to generate multiple-choice questions and reinforce learning. This step by step proof of concept module provides a scalable solution for training practitioners globally. It shows potential for improving education and assessment in interventional pain procedures, warranting further research to validate its efficacy.

## Declaration of competing interest

The authors declare that they have no known competing financial interests or personal relationships that could have appeared to influence the work reported in this paper.

